# Some Cases of Diabetes

**Published:** 1910-11-12

**Authors:** Nestor Tirard


### Hospital Clinics.

### SOME CASES OF DIABETES.

By NESTOR TIRARD, M.D., F.R.C.P.

A Lecture delivered at the Polyclinic, Chenies Street, W.C., on Monday, October 10, 1910.

Gentlemen,—The subject I propose to speak about to-day is diabetes. For a post-graduate lecture it appears to me to be fitting that attention should be mainly concentrated upon the clinical practical features, rather than upon merely theoretical considerations.

It is quite true that the subject of diabetes is one which has recently been ardently studied from various points of view. Ever since Claude Bernard's experiment upon the puncture of the floor of the fourth ventricle, and its influence in producing glycosuria, numerous physiologists and pathologists have sought, and still seek, to find some explanation of the causation of this disease. On the other hand, since the recognition of glucose in the urine as a prominent feature of this disease, we know that numerous efforts have been made to cure the disease by influencing the diet, diminishing the quantity of carbohydrate, in the hope of reducing the elimination of the glucose. Efforts have also been made from the commercial side to produce some form of diet which shall be free from sugar and reputedly free from carbohydrate, and, in addition, free from any danger of increasing the amount of sugar being formed.

### Diet Results.

The records in regard to diabetes fall under two heads: first, those resulting from physiological and clinical and pathological experiments—not always on the human being; and, secondly, those recording the benefits produced by some particular form of diet. These records, I think it is charitable to assume, often indicate a sanguine temperament, a temperament which is perhaps anxious to run into print before waiting for full investigation. It is certain that in hospital work we do not find confirmation of the benefits set forth as resulting from these various diets. You will gather from this that I do not propose to recommend any particular form of diet; I propose to employ our time to-day by keeping strictly to clinical considerations and observations, and to what appears to me to be important in the treatment of the individual.

### The Cases Cited.

The cases to which I shall refer in detail have been in my wards under close observation, and the notes I shall present have been furnished by many helpers—clinical pathologists, clinical clerks, ward sisters, etc., to all of whom I am much indebted. I shall also have occasion to refer briefly to cases which I have seen in consultation, and in regard to which it is not possible to feel on such certain ground. I would also mention that in this consideration of diabetes I am including only the saccharine form. During the summer session I had two well-marked cases of diabetes insipidus, but although much trouble was taken with them, they taught very little that was helpful, either as to the method of controlling the disease or as to its causation.

### The Urine Output.

For the first consideration I want to put before you I am indebted to a former student; it is a perfectly simple one. Is it ever desirable to attempt to influence the daily output of urine?—that is, to influence it directly by cutting off supplies. But for the fact that I have recently been asked this question I should hardly have thought it necessary to refer to it here. If we consider that one of the features of diabetes is a large increase in the daily amount of urine passed, and if we adopt a sort of rule-of-thumb method of treatment to reduce the amount which the patient takes, and so reduce the amount which the patient passes, we shall very soon arrive at disastrous results. As we all know, sugar is a diuretic, and it is impossible merely by cutting off the amount of liquid taken to reduce usefully to any great extent the amount of urine, and certainly the amount of glucose, that is being passed. We may reduce the amount of urine to some extent, but if we do so at the expense of the tissues, we lead to dehydration of the tissues and wasting; we see this in connection with any other quick withdrawal of fluid from the body, as, for example, in diarrhœa. No only so, but we increase the discomfort by provoking intense thirst. it is sufficient to mention this point and to pass it over at once.

### The Question of Decreasing the Sugar.

The second question is much more important: is it possible, and if possible is it desirable, in a well-established case of diabetes to attempt to arrest the glycosuria? Now, that must be answered somewhat at length. All cases of glycosuria are not cases of diabetes in the sense in which we are now using the term. In the dietetic glycosurias, in which the patient has been in the habit of taking large quantities of sugar as a dietary indulgence, it is perfectly possible to reduce the sugar to *nil*. I do not know that I need give you instances, but I may mention a case of an individual who appeared to be examined for life assurance, who took daily about three tablespoonfuls of marmalade. As he passed sugar, the decision was postponed until he mended his ways in regard to the marmalade, no other change having been made in his diet. It is possible in the same way to reduce or arrest the elimination of sugar in gouty cases. I do not think I need dwell upon this at any length. But in my experience it is not so easy, and I think it is generally undesirable to keep the urine free from sugar in diabetic cases. In some of the text-books on this subject, notably in von Noorden's, you will find it mentioned as though it is quite easy, by modifying the diet and giving opium, always to reduce the sugar to *nil*. In one of the cases I shall mention this was done for a time; but that case stands by itself, for although the same measures were taken subsequently in other cases traces of sugar still persisted. The case in which we did succeed was that of a girl aged twenty-four, who was under observation in the hospital from the early part of January until late in April. During the early part of that time she was passing a large amount of sugar, over 2,700 grains of it per day. This chart indicates how the sugar came down as a result of treatment, partly dietetic, and partly drug. You see there is a rise, which I will explain presently, and then the sugar disappears. After another short rise it disappeared again, and for a time we were very happy. But about April in the following year the patient came back to the hospital with the same saccharine condition of the urine, and this time we were totally unable to remove the traces of sugar. The chart exhibited also indicates the weight of the patient in terms of stones. There is a drop in the weight when the sugar is large in amount, and with the disappearance of the sugar the weight goes up, so that when she left the hospital she weighed nine stone, whereas in the early stage she weighed only a little over seven stone. In general, while the weight increases we can feel satisfied that most of the symptoms are doing well, even though some sugar may persist.

### An Illustrative Case.

I have here the record of a male patient, aged twenty-seven, who had much sugar at the commencement, but the quantity fell very speedily, though it could not be made to disappear from the urine altogether. We noticed a similar increase of weight with the reduction of sugar. Then there was a considerable rise in the amount of sugar, and the weight fell. When the patient left he weighed only a little over six stone, and was still passing 2,000 to 2,500 grains of sugar in the day. The treatment of those two patients was essentially the same. Where it differed was, that in the second case we adopted one or two of the newer forms of treatment, with temporary success. That I shall have to refer to later. It is not, therefore, always possible, even if it is desirable, to eliminate all the sugar from the urine; and further, it is often undesirable even to attempt its complete removal. Many times in hospital, and occasionally outside the hospital, one has seen cases in which the sudden arrest of sugar and carbohydrate in the diet has been immediately followed by the onset of coma. The dietetic modification should always be brought about by degrees.

### Complications.

Assuming the undesirability to produce suddenly any great reduction in the amount of sugar, what can we do to relieve the more prominent symptoms or the chief complications? I say relieve, because at the present time I fear that is all we can hope for. We can do a good deal for the relief of certain symptoms; in many cases we can postpone the end. You know that the common symptoms are wasting, dry skin, depression. These symptoms may be overlooked, the patient may not trouble about them, but they are there, even in the occasional cases, where the attention is attracted in the first place by some more serious symptoms. Of the complications it is of course natural that I should first deal with renal complications. Now and again, either in hospital work, or still more, when examining students, one finds that the presence of albuminuria, for instance, and its influence on the condition of urine is not quite appreciated. Another reason that I have for putting the renal complications prominently forward is, that in June this year we had a patient admitted unconscious, in whose case it was for some time a matter of doubt as to whether the symptoms were primarily uræmic or primarily diabetic. Other complications are cardio-vascular changes. The mere weakness of the heart does not very much matter as a rule. It may occasionally lead to syncope as apart from coma. Weakness of the heart muscle is not nearly so important as the alteration in the character of the vessels, for the vascular changes lead either to the development of gangrene or possibly to the formation of perforating ulcers. As to the nervous complications, the weariness, the sense of fatigue, and the depression may perhaps be accounted as symptoms. But there are also more definite nervous complications, loss of knee jerks and forms of neuritis, and the condition known as "air-hunger." I think this last must be put to the credit of the nervous side of the case. There is much discussion with regard to it, as to whether the influence is through the carbonic acid or through some other blood change. The thing that is certain is that with air-hunger there is a sense of urgent dyspnœa, which appears to act primarily through the nervous system. I think we also have to place on the side of the nervous system the cases in which there is severe abdominal pain. There have been several cases recorded of diabetes with vomiting and abdominal pains, as prominent symptoms. I have had two under my own observation in whom the symptoms, shortly before death, strongly simulated appendicitis or some other acute abdominal condition.

*(To be continued.)*

